# Coupling a Simple and Generic Membrane Fouling Model with Biological Dynamics: Application to the Modeling of an Anaerobic Membrane BioReactor (AnMBR)

**DOI:** 10.3390/membranes14030069

**Published:** 2024-03-20

**Authors:** Boumediene Benyahia, Amine Charfi, Geoffroy Lesage, Marc Heran, Brahim Cherki, Jérôme Harmand

**Affiliations:** 1 Laboratoire d’Automatique de Tlemcen, Faculté de Technologie, Université de Tlemcen, P.O. Box 230, Tlemcen 13000, Algeria; brahim.cherki@univ-tlemcen.dz; 2 UR ABTE, Département de Génie Chimique Génie des Procédés, IUT GON, Université de Caen Normandie, 14032 Caen, France; amine.charfi@unicaen.fr; 3 Institut Européen des Membranes, 300 Avenue du Prof. Emile Jeanbrau, 34090 Montpellier, France; geoffroy.lesage@umontpellier.fr (G.L.); marc.heran@umontpellier.fr (M.H.); 4 LBE-INRAE, 102 Avenue des Étangs, 11100 Narbonne, France; jerome.harmand@inrae.fr

**Keywords:** anaerobic membrane bioreactor, identification, MBR modeling, membrane fouling, SMP, wastewater treatment

## Abstract

A simple model is developed for membrane fouling, taking into account two main fouling phenomena: cake formation, due to attached solids on the membrane surface, and pore clogging, due to retained compounds inside the pores. The model is coupled with a simple anaerobic digestion model for describing the dynamics of an anaerobic membrane bioreactor (AnMBR). In simulations, we investigate its qualitative behavior: it is shown that the model exhibits satisfying properties in terms of a flux decrease due to membrane fouling. Comparing simulation and experimental data, the model is shown to predict quite well the dynamics of an AnMBR. The simulated flux best fits the experimental flux with a correlation coefficient $r^{2}=0.968$ for the calibration data set and $r^{2}=0.938$ for the validation data set. General discussions are given on possible control strategies to limit fouling and optimize the flux production. We show in simulations that these strategies allow one to increase the mean production flux to 33 L/(h·m^2^),whereas without control, it was 18 L/(h·m^2^).

## 1. Introduction

The anaerobic membrane bioreactor (AnMBR) is an interesting wastewater treatment technology, which couples anaerobic digestion treatment of organic pollutants with a physical separation between the sludge and liquid which improves the purification of the produced effluent. To reach an optimal treatment efficiency, it is crucial to control both the biological and the separation processes. Thus, it is important to model biological dynamics and couple them to a membrane filtration model to predict membrane fouling, which remains, by far, the main drawback of MBR technology [1,2,3].

Recent work established that models developed to describe the conventional activated sludge process (Activated Sludge Models (ASM) [4]) and anaerobic digestion (Anaerobic Digestion Model N.1 [5]) can be used to describe MBR dynamics when slightly modifying the model parameters [6]. However, such models were developed above all for continuous and homogeneous reactors and are not able to account for specific components such as Soluble Microbial Product (SMP) dynamics that are known to play an important role in membrane fouling [7,8,9,10,11]. In a conventional bioreactor, the matter recycling (due for instance to biomass mortality) is not necessarily taken into account, because it can usually be neglected with respect to the dilution rate. In MBRs, this hypothesis no longer holds, and variables describing the dynamics of certain classes of molecules, such as the SMP produced during biomass growth and mortality, must be added to the model. For anaerobic systems, simpler models than the ADM1 have been coupled to SMP dynamics. For instance, we proposed an extension of the two-step anaerobic digestion model (AM2) [12], in order to include their dynamics [13].

Regarding membrane fouling, many models have been proposed such as the resistance-in-series model including the kinetics of SMP and extra-cellular polymeric substances (EPS) [14] (a brief review on the Resistance-in-Series Model in MBRs was highlighted in [15]), data-driven approach with membrane fouling control [16], machine learning techniques for potential application in MBR [17], models based on a sectional approach [18] and on the fractal geometry to describe the fouling cake permeability [19], models based on the local pressure and flux variation leading to the uneven fouling cake up on the membrane surface [20], and physical models which have been proposed to study fundamental membrane properties [21,22,23]. Simpler models are proposed to describe fouling as the result of only the cake formation mechanism or adding pores blocking phenomenon due to soluble matter [24]. These models are purely physical ones; they describe the dynamics of abiotic membrane parameters and completely neglect biological dynamics even if some authors, such as [25], proposed to combine them to describe fouling in MBRs. Thanks to advances in computer science, alternatives to mathematical modeling have been introduced to model membrane fouling. In [26], an artificial neural network was used to predict the transmembrane pressure of a large pilot-scale AnMBR reactor and to provide a suitable model for intelligent control purposes. A prediction of the membrane fouling status before reaching a critical condition using neural network modeling was carried out in [27], with the aim to extend the membrane life.

The present study aims to describe the dynamics of the entire MBR process by coupling the biological process dynamics and the membrane fouling models, and while numerous integrated models have been proposed for aerobic MBRs (cf. for instance [14,28,29]), few have been developed for anaerobic MBRs. In [30], authors developed a mathematical model for MBR, by considering together reversible and irreversible fouling. Mixed liquor suspended solids were assumed to be the major components of the reversible fouling layer, and dissolved organic matter is thought to be responsible for the long-term irreversible fouling. Ref. [31] proposed a filtration model based on the resistance-in-series model and was able to reproduce the filtration process of a Submerged AnMBR (cake layer build-up and consolidation during filtration; membrane scouring by biogas sparging; removal of cake layer by back-flushing; and irreversible fouling consolidation). This model was validated in the long-term under different operating conditions, using data obtained from a SAnMBR demonstration plant [32]; while such models have high prediction capabilities, they are usually too complicated for being used for control purposes. Ref. [33] proposed a model for a Submerged MBR, with slow–fast dynamics, and they used this structure for the parameter estimation procedure. Thereafter, the model is used to develop a nonlinear predictive control.

In [13], the AM2b model was specifically proposed for control purposes. It is a simple model which describes only the two main biological processes of the anaerobic digestion while including the SMP dynamics. In the first step (acidogenesis), the acidogenic bacteria $X_{1}$ consume the organic substrate $S_{1}$ to produce volatile fatty acid $S_{2}$ (VFA), *SMP* and $CO_{2}$, while in the second step (methanogenesis), the methanogenic population $X_{2}$ consumes VFA and produces *SMP*, methane and $CO_{2}$. However, this model was not coupled with a membrane fouling model to completely describe the dynamics of an entire AnMBR in the simplest way that we can think of for control synthesis purposes.

To summarize the state of the art about AnMBR models, one may say that available fouling models are either not coupled to the biological models, or they are too complicated to be used in process control [34]. This is precisely the aim of the present paper, where the novelty is to propose a simple and generic membrane fouling model of which the usefulness is illustrated in coupling it with biological model to completely describe an AnMBR and to develop optimization tools and strategy. In addition, we consider only two fouling mechanisms for the membrane model, which depend on the biological model outputs (measurements of the biological model are inputs for the fouling model). Contrary to several research studies on fouling modeling, we assume that the total membrane open area would decrease during the filtration process as well as after repeated filtration/cleaning cycles, proportionally to the fouling layer mass developed on the membrane surface and within its pores, resulting in irreversible fouling leading to partial membrane degeneration. It is described in a very general way as a decreasing function of deposited matters onto the membrane surface and retained into membrane pores.

The advantage of the model we propose is that it is a generic model, with enough processes and parameters to reproduce just about all the data from all membrane filtration processes. It is a good candidate model, more “reproductive” than “descriptive”, with some parameter values guided by experimental data.

The paper is organized as follows. First, we present and discuss the assumptions used to build the membrane model. Then, the dynamic equations of the model are presented with respect to the specific functioning phases considered: filtration and cleaning phases. Then, simulation results are presented in order to study the qualitative properties of the model. To illustrate the easiness with which it may be coupled to a biokinetic model, the fouling model is then coupled with the AM2b model and confronted to experimental data. Thereafter, some techniques for fouling control are discussed and simulated. Finally, conclusions and perspectives are formulated.

## 2. Mathematical Equations of the Proposed Membrane-Fouling Model

### 2.1. Model Development

Ref. [18] proposed a membrane fouling model including an explicit relationship between the mass of solid matter attached onto the membrane and the flux going through this membrane. The dynamics of the solid attachment to and detachment from the membrane were related to the filtration flux. Using a resistance-in-series model, they considered the total fouling resistance to be caused both by the pore-clogging resistance due to the solute deposition inside the membrane pores, the dynamic sludge-film resistance and the stable sludge-cake resistance. However, the proposed model is not suitable for automatic control purposes since it is too complicated. The aim of the present section is to simplify this model to come up with a very simple fouling model while keeping realistic hypotheses. The idea is to include a feedback of the decreasing flux due to membrane fouling into the actual output flow rate $Q_{out}$ leaving the MBR. In other words, we propose to consider $Q_{out}$ as a decreasing function of the total mass solids attached onto the membrane surface and of the solute (as SMP) deposited inside the pores. As recalled in the introduction, as a matter of fact, many studies in the literature agreed that SMP have a crucial role in the membrane fouling, especially in pore clogging [7,8,9,10,11].

**Figure 1:**
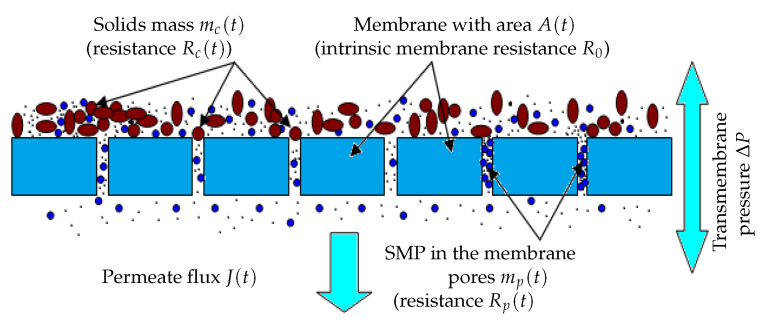
Membrane fouling by cake formation and pores clogging.

- **Fouling mechanisms**
  It is well known that the fouling dynamics are different depending on the fouling mode considered, namely pore constriction, cake formation, complete blocking and intermediate blocking [22]. In our simple model, we consider only the two main membrane fouling mechanisms, as defined in [21] (see Figure 1):
  - The first one is caused by the mass $m_{c}\left(t\right)$ of solids which attach onto the membrane surface, also called cake formation or cake fouling. According to the particles concentration and solids attachment rate, particles are retained leading to a decrease in the filtering area of the membrane.
  - The second is due to the mass of particles $m_{p}\left(t\right)$ retained inside the membrane pores as SMP, called hereafter pore constriction. Their size may be smaller than the pore sizes, and they are known to progressively clog the membrane pores. This phenomenon typically reduces the porous area of the membrane.

The proposed modeling approach allows us to decouple dynamically the different fouling mechanisms (solids attached onto the membrane vs. SMP clogging the pores). Using a resistance-in-series model based on Darcy’s law, the membrane fouling model for plants operating at constant transmembrane pressure (TMP) is thus given by (1):

(1) $$J\left(t\right)=\frac{Q_{out}\left(t\right)}{A(t)}=\frac{\Delta P}{\mu \left(R_{0}+R\left(t\right)\right)},$$

where $Q_{out}\left(t\right)$ is the output flow rate, A(t) is the membrane surface area, ΔP is the transmembrane pressure (assuming constant), μ is the permeate viscosity, $R_{0}$ is the intrinsic membrane resistance, and R(t) is the fouling resistance given by (2)

(2) $$R\left(t\right)=R_{c}\left(t\right)+R_{p}\left(t\right),$$

where $R_{c}\left(t\right)$ and $R_{p}\left(t\right)$ are the cake and the pore clogging resistances, respectively.

**Figure 2:**
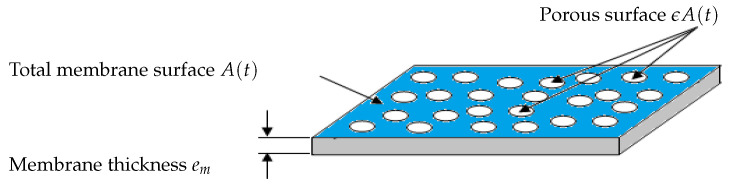
Schematic representation of the total membrane surface *A* and the total porous surface ϵA.

**Figure 3:**
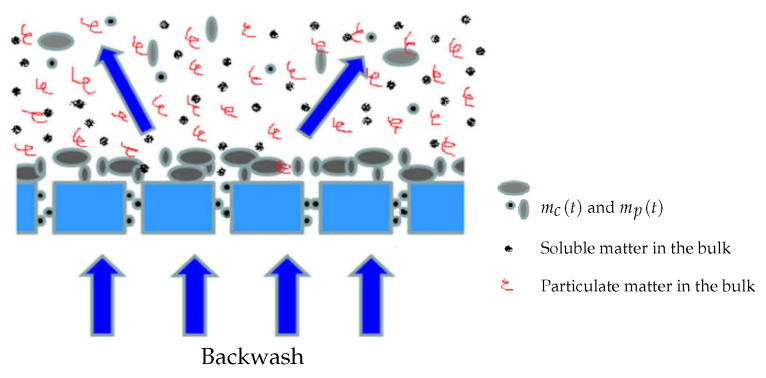
Detached matter by relaxation or backwash is neglected in the bulk liquid.

- **Models of membrane resistance and membrane area**
  $R_{c}\left(t\right)$ and $R_{p}\left(t\right)$ are typically dependent on masses $m_{c}\left(t\right)$ and $m_{p}\left(t\right)$, respectively, and are modeled by (3) (adapted from [14])
  (3) $$R_{c}\left(t\right)=\alpha \frac{m_{c}\left(t\right)}{A(t)},\;R_{p}\left(t\right)=\alpha^{\prime }\frac{m_{p}\left(t\right)}{\epsilon A(t)},$$
  where α and $\alpha^{\prime }$ are the specific resistances, and ϵA (0<ϵ<1) is the porous area which is a fraction of the total useful surface area *A* (see Figure 2 for a flat sheet membrane for instance).
  Contrary to several studies on fouling modeling, we consider that the total filtering membrane surface area A(t) is not constant during a filtration period nor after several filtration/cleaning cycles. It is described by (4) in a very general way as a decreasing function of $m_{c}\left(t\right)$ and $m_{p}\left(t\right)$ as follows:
  (4) $$A\left(t\right)=\frac{A_{0}}{1+\frac{m_{c}\left(t\right)}{\sigma }+\frac{m_{p}\left(t\right)}{\sigma^{\prime }}},$$
  where $A_{0}$ is the initial membrane surface, and σ and $\sigma^{\prime }$ are parameters used to model the contribution of $m_{c}$ and $m_{p}$ to the surface reduction. Such a function is well adapted if we assume that the total useful filtration area is composed of two parts: a filtering surface and a porous surface. If $m_{p}\left(t\right)$ increases, then the porous surface decreases, leading to the total loss of A(t) even if the cake fouling is not yet significant. Likewise, if $m_{c}\left(t\right)$ increases then the filtering surface decreases because attached particles may prevent the flux from circulating freely, even if the pore-clogging fouling reaches its equilibrium or if it is not yet significant. In short, the membrane surface A(t) decreases when $m_{p}\left(t\right)$ and/or $m_{c}\left(t\right)$ increase (mathematically, A(t) tends to zero as $m_{c}\left(t\right)$ and/or $m_{p}\left(t\right)$ tend to infinity).
  Function (4) is also able to model the fact that the initial filtering surface $A_{0}$ is not totally recovered after backwash or chemical cleaning. Theoretically, in Equation (4), if $m_{c}\left(t\right)=0$ and $m_{p}\left(t\right)=0$ when we operate the MBR plant for the first time, or after each perfect backwash of the membrane, then the area A(t) is equal to its initial value $A_{0}$. However, in practice, after each membrane backwash or cleaning, there is small remaining quantities of $m_{c}\left(t\right)$ and $m_{p}\left(t\right)$ which are not detached, causing progressively an irreversible fouling effect. In the long term, the surface A(t) continuously decreases, leading to the membrane degeneration.

- **Models of attached solids on the membrane surface and blocked SMP into pores**
  Both compounds $m_{c}\left(t\right)$ and $m_{p}\left(t\right)$ have their own dynamics: they increase during the filtration phase and decrease during the relaxation (or backwash) phase. Since it is assumed that the mixed liquor is homogeneous, we assume that all soluble components ($S_{T}=\sum S_{i}$, i=1,2,… and *SMP*) and particulate components ($X_{T}=\sum X_{i}$, i=1,2,…) may contribute, at different degrees, to the membrane fouling by cake formation (solids attachment). Thus, the dynamic of the mass $m_{c}\left(t\right)$ can be described by (5)
  (5) $${\dot{m}}_{c}=Q_{out}\left(C_{s}S_{T}+C_{x}X_{T}+C_{smp}SMP\right),$$
  where $C_{s}$, $C_{x}$ and $C_{smp}$ are weighting parameters used to model the contribution and the rate of each variable to the cake formation. In practice, they must be adjusted using calibration data (see the experimental results Section 4).
  The membrane has selective rejection: particulate components (biomass) and large solute compounds (as macro-molecules of *SMP*) are totally retained by the membrane (their size being supposed to be greater than the pores diameter), while part of the solute components (substrates and a fraction of *SMP*) go through the membrane without retention (their size is assumed to be smaller than the pores diameter). We propose the following dynamic model (6) for the pores clogging by $m_{p}\left(t\right)$:
  (6) $${\dot{m}}_{p}=Q_{out}\left(\beta_{1}SMP+\beta_{2}S_{T}\right),$$
  where $\beta_{1}$ is a parameter used to calibrate the rate of pores clogging, by the fraction of *SMP* leaving the bioreactor, while $\beta_{2}$ is used to model the contribution of others solute substrates to the pores clogging.
  On the other hand, no back-diffusion of $m_{c}\left(t\right)$ and $m_{p}\left(t\right)$ to the bulk solution is considered: we assume it is negligible with respect to the remaining attached and blocked matter.

- **Additional hypothesis: There is no biomass growth on the membrane surface, and detached solids do not affect matter concentration in the bulk liquid.**
  For simplicity, we assume that the biological growth of the attached biomass on the membrane (as well as in the pores) is neglected. This hypothesis is justified by the fact that backwash or relaxation periods arise quite often. In addition, we assume that if there are detached quantities of $m_{c}\left(t\right)$ and $m_{p}\left(t\right)$ during relaxation, which return into the bioreactor, they can be neglected with respect to their corresponding concentrations in the bulk liquid (see Figure 3). In many operated MBR, detached matter by backwash is not returned into the reaction medium and is rejected elsewhere. Finally, both fouling mechanisms are considered to be partially irreversible, but at different degrees, i.e., fouling by pores clogging is more irreversible than fouling by cake formation.

### 2.2. Fouling Model for the Filtration Phase

Starting from the previous equations and hypotheses, the complete fouling model for the filtration phase (ΔP>0) is given by Equations (7)–(11). The output flow rate $Q_{out}$ is a decreasing function: after a certain period of functioning when the permeate flux has dramatically decreased, the process must be stopped and membrane cleaning must be performed.

(7) $$\begin{array}{ccc} {\dot{m}}_{c} & = & Q_{out}\left(C_{s}S_{T}+C_{x}X_{T}+C_{smp}SMP\right), \end{array}$$

(8) $$\begin{array}{ccc} {\dot{m}}_{p} & = & Q_{out}\left(\beta_{1}SMP+\beta_{2}S_{T}\right), \end{array}$$

(9) $$\begin{array}{ccc} R & = & \alpha \frac{m_{c}}{A}+\alpha^{\prime }\frac{m_{p}}{\epsilon A}, \end{array}$$

(10) $$\begin{array}{ccc} A & = & \frac{A_{0}}{1+\frac{m_{c}}{\sigma }+\frac{m_{p}}{\sigma^{\prime }}}, \end{array}$$

(11) $$\begin{array}{ccc} Q_{out} & = & J\cdot A=\frac{\Delta P\cdot A}{\mu \left(R_{0}+R\right)}. \end{array}$$

In Equation (7) the dynamics of $m_{c}\left(t\right)$ are proportional to the total soluble ($S_{T}$ and SMP) and particulate matter ($X_{T}$) deposited onto the membrane surface. If we can measure separately the components of $S_{T}$ ($S_{1}$, $S_{2}$, ...) and/or those of $X_{T}$ ($X_{1}$, $X_{2}$, ...), where each one contributes differently to the cake formation, then Equation (7) can be written by (12)

(12) $${\dot{m}}_{c}=Q_{out}\left(\sum C_{si}S_{i}+\sum C_{xj}X_{j}+C_{smp}SMP\right),$$

where $C_{si}$ (i=1,2,…) and $C_{xj}$ (j=1,2,…) are weighting coefficients.

In Equation (8), the dynamics of $m_{p}\left(t\right)$ are essentially proportional to the fraction $\beta_{1}\cdot SMP$ crossing the membrane and blocked into the pores (macro-molecules of *SMP* are retained by the membrane, see [13]). However, after a long enough filtration time, porosity decreases, and pores clogging is also assumed to be dependent on small deposited quantities of $S_{T}$, but less significantly than *SMP*. This is modeled by $\beta_{2}\cdot S_{T}$, with $\beta_{2}$ chosen lower than $\beta_{1}$.

The dynamics of $m_{c}\left(t\right)$ and $m_{p}\left(t\right)$ depend on the values of variables $S_{T}$, $X_{T}$, SMP and $Q_{out}$. For a bioreactor using organic tubular membrane with tangential crossflow (the case of our studied AnMBR, see Section 4) and when the system operates at steady state, $S_{T}$, $X_{T}$, SMP and $Q_{out}$ reach their equilibria, and thus, $m_{c}$ and $m_{p}$ converge to their steady state values, where their accumulation values are constant. Consequently, the fouling resistance R(t) reaches its equilibrium corresponding to the maximum fouling. This interpretation corresponds to reality since membrane fouling increases with time. For a long enough filtration time, there is no longer attachment of matter onto the membrane, because of the cake layer already formed: this equilibrium corresponds to a functioning mode in which detachment by shear forces compensates the attachment of matter by permeation forces. In fact, the tangential flow of the feed solution in a cross-flow system creates turbulence and consequently shear forces near by the membrane, which would foster the back diffusion of foulants polarized near to the membrane surface and the detachment of deposited foulants. These phenomena would reduce membrane fouling. During the filtration process, foulant transport is controlled by both convective forces which attract them to the membrane and the shear forces which repulse them away. Those forces are responsible for the set of an equilibrium which leads to the stabilization of the deposit mass.

### 2.3. Fouling Model for the Relaxation (or Backwash) Phase

To clean the membrane by backwash, the feed of the MBR is stopped, and the transmembrane pressure is inverted (ΔP<0), so that permeate flow backs into the feed, lifting the fouling layer from the pores and the surface of the membrane. In certain cases, MBR are operated with a relaxation period instead of a backwash. In others terms, the flux is simply stopped (ΔP=0) for a given short time allowing the natural detachment of matters and particles, which can be modeled by Equations (13) and (14), with ω and $\omega^{\prime }$ positive constants to be adjusted with respect to experimental data, $m_{c_{irr}}$ and $m_{p_{irr}}$ positive quantities to model irreversible fouling after cleaning operation (quantities of irreversible $m_{c}$ and $m_{p}$). For instance, we assume that we have x% of irreversible fouling after relaxation or backwash, which means that after membrane cleaning, x% of $m_{c}$ and $m_{p}$ reached at the end of the previous filtration period is irreversible.

(13) $$\begin{array}{ccc} {\dot{m}}_{c} & = & -\omega m_{c}+m_{c_{irr}}, \end{array}$$

(14) $$\begin{array}{ccc} {\dot{m}}_{p} & = & -\omega^{\prime }m_{p}+m_{p_{irr}}. \end{array}$$

The relaxation (or backwash) time is neglected compared to the filtration time, and it is expected that after this period, $m_{c}\left(t\right)$ and $m_{p}\left(t\right)$ are approximately equal to their initial values. However, there is always a certain quantity of attached matter which may remain on the membrane surface and/or blocked inside the pores, yielding irreversible fouling. Using the hypotheses discussed above, one can also neglect concentrations of detached matter returning to the reactional medium. It is important to emphasize that all (or part of) model parameter values must be adjusted using experimental data. In the next section, we choose arbitrary values of parameters for properties numerical investigation of the systems (7)–(11), (13) and (14).

## 3. Simulation Results

### 3.1. Coupling the Membrane Fouling Model with the AM2b Model

**Figure 4:**
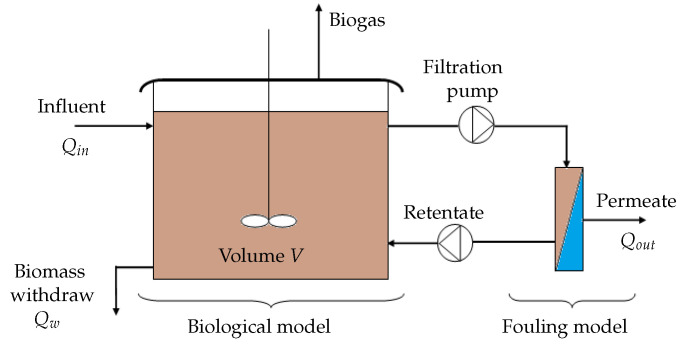
Schematic representation of the proposed AnMBR model.

The proposed integrated model combines a biological anaerobic model and the fouling model for an homogeneous bioreactor as illustrated in Figure 4. For the biological compartment, the anaerobic digestion model is not specified here. Unless the effects of SMP on fouling are neglected as in [6], neither the ADM1 model [5] nor the AM2 model [12] are good candidates since they do not include SMP dynamics. Instead, we suggest using the AM2b model [13] which includes SMP dynamics and that has been precisely developed for control purposes. In any case, to couple it with the proposed fouling model, soluble and particulate matters must be related to state variables of the AM2b model. In addition, two functioning phases are considered: filtration and relaxation or backwash. By convention, ΔP>0 holds for filtration; ΔP=0 holds for relaxation; and ΔP<0 holds for backwash.

### 3.2. Investigating the Qualitative Behavior of the Model

In this section, we investigate numerically the model dynamics consisting of the coupling of Equations (7)–(11), (13) and (14) with the anaerobic digestion model AM2b [13]. The AM2b model was developed to describe anaerobic digestion as a two step process, including dynamics of SMP. In the first step (acidogenesis), the acidogenic bacteria $X_{1}$ consume the organic substrate $S_{1}$ to produce volatile fatty acid $S_{2}$ (VFA), SMP and $CO_{2}$, while in the second step (methanogenesis), the methanogenic population $X_{2}$ consumes VFA and produces SMP, methane and $CO_{2}$.

The integrated model is given by Equations (15) and (16), and it has been running in simulation using the ode45 function of MatLab, with the set of parameter values given in Table A1. Numerical simulations are performed for two cycles of filtration/relaxation and for three distinct combinations of parameters values $C_{s}$, $C_{x}$ and $C_{smp}$ (for simulation, their values are equal to 0.1, 0.4 or 0.7). Quantities of soluble and particulate matters $C_{s}S_{1}$, $C_{s}S_{2}$, $C_{smp}SMP$, $C_{x}X_{1}$ and $C_{x}X_{2}$ attracted by permeation forces $Q_{out}/V$ are assumed to be fully attached to the membrane surface. We consider that the relaxation period (5 min) is negligible compared to the filtration period (2 h). During the relaxation or backwash period, particulate and soluble matters partially re-injected into the bulk are taken into account in the model (16) through the parameters $C_{s}^{\prime }$, $C_{x}^{\prime }$ and $C_{smp}^{\prime }$. Between two cleaning cycles, the bulk volume V(t) is constant thanks to the volume dynamics equation and the flux balance given in model (15). When $Q_{out}$ decreases, then $Q_{in}$ should decrease or $Q_{w}$ should increase or both should vary. Experimentally, both $Q_{in}$ and $Q_{w}$ are judiciously fixed in such a way that we obtain an optimal ratio of organic matters (COD)/mixed liquor volatile suspended solids concentration (MLVSS)/day.

It should be noticed here that the precise adjustment of the filtration/relaxation (or backwash) periods in MBRs is an open problem of control where an optimal solution must be searched for. This problem is usually solved in applying short filtration sequences followed by relaxation/backwash periods in such a way that the clogging is very limited. However, it must be underlined that such sequences are not optimized and are probably quite far from an optimal.

**Coupled model for the filtration phase:**

(15) $$\begin{array}{ccc} {\dot{X}}_{1} & = & \left(\mu_{1}\left(S_{1}\right)+\mu_{smp}\left(SMP\right)-k_{d1}-\frac{Q_{w}}{V}-\frac{Q_{out}}{V}C_{x}\right)X_{1},\\ {\dot{X}}_{2} & = & \left(\mu_{2}\left(S_{2}\right)-k_{d2}-\frac{Q_{w}}{V}-\frac{Q_{out}}{V}C_{x}\right)X_{2},\\ {\dot{S}}_{1} & = & \frac{Q_{in}}{V}S_{1in}-\left(\frac{Q_{out}}{V}+\frac{Q_{w}}{V}\right)S_{1}-k_{1}\mu_{1}\left(S_{1}\right)X_{1}-\frac{Q_{out}}{V}C_{s}S_{1},\\ {\dot{S}}_{2} & = & \frac{Q_{in}}{V}S_{2in}-\left(\frac{Q_{out}}{V}+\frac{Q_{w}}{V}\right)S_{2}-k_{3}\mu_{2}\left(S_{2}\right)X_{2}+\left(k_{2}\mu_{1}\left(S_{1}\right)+b_{2}\mu_{smp}\left(SMP\right)\right)X_{1}\\ \; & \; & -\frac{Q_{out}}{V}C_{s}S_{2},\\ \dot{SMP} & = & -\left(\beta \frac{Q_{in}}{V}+\left(1-\beta \right)\frac{Q_{w}}{V}\right)SMP+\left(b_{3}\mu_{1}\left(S_{1}\right)+k_{d1}-b_{1}\mu_{smp}\left(SMP\right)\right)X_{1}\\ \; & \; & +\left(b_{4}\mu_{2}\left(S_{2}\right)+k_{d2}\right)X_{2}-\frac{Q_{out}}{V}C_{smp}SMP,\\ {\dot{m}}_{c} & = & Q_{out}\left(C_{s}S_{T}+C_{x}X_{T}+C_{smp}SMP\right),\\ {\dot{m}}_{p} & = & Q_{out}\left(\beta_{1}SMP+\beta_{2}S_{T}\right),\\ R & = & \alpha \frac{m_{c}}{A}+\alpha^{\prime }\frac{m_{p}}{\epsilon A},\\ A & = & \frac{A_{0}}{1+\frac{m_{c}}{\sigma }+\frac{m_{p}}{\sigma^{\prime }}},\\ Q_{out} & = & J\cdot A=\frac{\Delta P\cdot A}{\mu \left(R_{0}+R\right)},\\ Q_{in} & = & Q_{out}+Q_{w},\\ \dot{V} & = & Q_{in}-Q_{out}-Q_{w},\\ X_{T} & = & X_{1}+X_{2},\\ S_{T} & = & S_{1}+S_{2}+SMP. \end{array}$$

**Coupled model for the relaxation (or backwash) phase:**

(16) $$\begin{array}{ccc} {\dot{X}}_{1} & = & \left(\mu_{1}\left(S_{1}\right)+\mu_{smp}\left(SMP\right)-k_{d1}+C_{x}^{\prime }\right)X_{1},\\ {\dot{X}}_{2} & = & \left(\mu_{2}\left(S_{2}\right)-k_{d2}+C_{x}^{\prime }\right)X_{2},\\ {\dot{S}}_{1} & = & -k_{1}\mu_{1}\left(S_{1}\right)X_{1}+C_{s}^{\prime }S_{1},\\ {\dot{S}}_{2} & = & -k_{3}\mu_{2}\left(S_{2}\right)X_{2}+\left(k_{2}\mu_{1}\left(S_{1}\right)+b_{2}\mu_{smp}\left(SMP\right)\right)X_{1}+C_{s}^{\prime }S_{2},\\ \dot{SMP} & = & \left(b_{3}\mu_{1}\left(S_{1}\right)+k_{d1}-b_{1}\mu_{smp}\left(SMP\right)\right)X_{1}+\left(b_{4}\mu_{2}\left(S_{2}\right)+k_{d2}\right)X_{2}+C_{smp}^{\prime }SMP,\\ {\dot{m}}_{c} & = & -\omega m_{c}+m_{c_{irr}},\\ {\dot{m}}_{p} & = & -\omega^{\prime }m_{p}+m_{p_{irr}}. \end{array}$$

**Figure 5:**
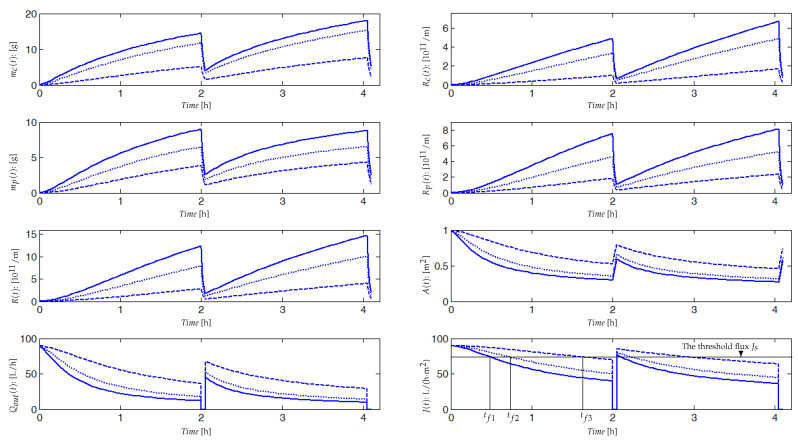
Simulation results of the membrane fouling model for both phases (filtration and backwash), with values of $C_{s}=C_{x}=C_{smp}$ equal to 0.1 (**—**); 0.4 (**---**); 0.7 (⬝⬝⬝⬝⬝). We have assumed that the mixed liquor is homogeneous and, to simplify the simulation, $S_{T}$, $X_{T}$ and SMP are assumed to contribute equally to the cake formation, then we chose $C_{s}=C_{x}=C_{smp}$ in each simulation.

Simulation results are reported in Figure 5, where we have plotted the dynamic evolution of the attached mass $m_{c}\left(t\right)$ on the membrane surface, the blocked soluble matter $m_{p}\left(t\right)$ (SMP in the majority) inside the pores, the fouling resistances $R_{c}\left(t\right)$, $R_{p}\left(t\right)$ and R(t), the output flow rate $Q_{out}\left(t\right)$, the permeate flux J(t) and the membrane surface A(t). Dynamic responses are simulated for three different combinations of parameters values $C_{s}$, $C_{x}$, $C_{smp}$ and $\beta_{1}$ to emphasize effects of deposited and blocked matter rates on the fouling dynamic. These rates depend on many parameters such as concentrations of soluble and particulate matters, characteristics of mixed liquor and its viscosity or still temperature and matters specific capability to contribute to fouling.

The trajectories of the main variables are plotted in the case of rapid and strong fouling due, for example, to a high concentration of solid matter. In such a case, if we define a threshold flux $J_{s}$, over which the process can operate, then the process will be stopped very often and be switched in relaxation or backwash phases (at $t_{f1}$ for the first operating cycle). Dashed plots correspond to a slower and softer fouling: the slower the fouling, the longer the time period ($t_{f3}$) during which the process may operate without switching in a relaxation or backwash mode. Such simulations show that $C_{s}$, $C_{x}$, $C_{smp}$ and $\beta_{1}$ may be adjusted to match a large range of experimental data.

During the first minutes of the filtration process, the fouling is fast and significant. All variables have fast dynamics (increasing or decreasing) at the beginning and then attain gradually their equilibrium. At steady state, quantities of the attached mass $m_{c}\left(t\right)$ on the membrane surface (around 15 g per 1 m^2^ of area), and the SMP mass blocked into the pores $m_{p}\left(t\right)$ (around 8 g) are sufficient to cause membrane fouling. Resistances of pore blocking $R_{p}\left(t\right)$ and cake formation $R_{c}\left(t\right)$ are within the order of $10^{11}$. The useful filtering surface area A(t), the output flow $Q_{out}\left(t\right)$ and the permeate flux J(t) decrease significantly, especially during the first minutes of filtration as is often the case in practice. During the relaxation (or backwash) phase, we have an exponential decrease in $m_{c}\left(t\right)$ and $m_{p}\left(t\right)$, with detachment from the membrane surface and pores (shear-induced diffusion). We notice that even after the relaxation (or the backwash), the permeate flux J(t) and the membrane area A(t) are not equal to their initial values (see Figure 5), because of the irreversible fouling taken into account in the model. From cycle to cycle, the total resistance R(t) has increasing equilibrium values. After several cycles, it will be necessary to clean chemically the membrane or to change it. Regarding these qualitative results, we claim that if we can accurately predict fouling (accumulated mass on the membrane and in the pores) using the developed model, then we can develop a control strategy to minimize fouling and run the membrane for a long time, using an optimal filtration–relaxation sequence.

## 4. Experimental Results

In this section, we are interested in the calibration of the fouling models (7)–(11), by using experimental data collected at the Center of Biotechnology of Sfax, Tunisia.

### 4.1. Pilot Plant (AnMBR) and Data Used for the Model Validation

**Figure 6:**
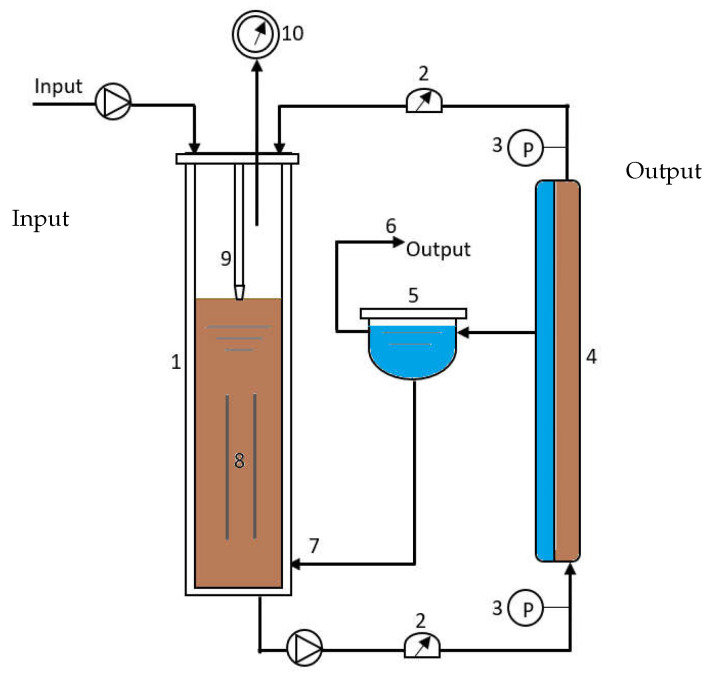
Schematic diagram of the AnMBR installed at CBS for the municipal and abattoirs wastewater treatment. 1: Anaerobic reactor, 2: Flow-meter, 3 Manometer, 4: Ultra-filtration membrane, 5: Permeate tank, 6: Permeate output, 7: Permeate recycling, 8: Column, 9: Tube, 10: Gas-meter.

**Figure 7:**
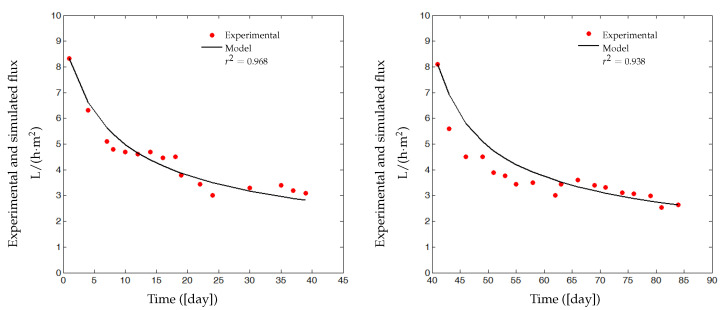
(**Left**): Data used to calibrate model parameters and simulated response of flux J(t). (**Right**): Validation of the model using a second set data. Operating conditions: pressure: 1.5 bar; temperature: 37 °C; cross-flow velocity 3 m/s (for more detail on experimental process and data see [35,36]).

The schematic representation of the pilot plan is shown in Figure 6. It was installed at the “Center of Biotechnology of Sfax”, Tunisia, and it was used for the treatment of municipal wastewaters. The system is composed of an anaerobic bioreactor coupled with an ultrafiltration organic tubular membrane module, with tangential crossflow and a filtering area of 1 m^2^ and 100 kDa cut-off. The working volume of the bioreactor is 50 L, and its temperature is maintained constant at 37 °C. The cross-flow velocity is fixed at 3 m/s, and the transmembrane pressure may vary until reaching 2 bars. The membrane was frequently chemically cleaned, almost every 45 days, in order to maintain an acceptable flux and avoid a critical clogging of the membrane (see Figure 7). The cleaning step was performed for 1 h at 35 °C and was followed by water cycle. For more details on the experimental process and analytical methods, the reader is referred to [35,36].

**Figure 8:**
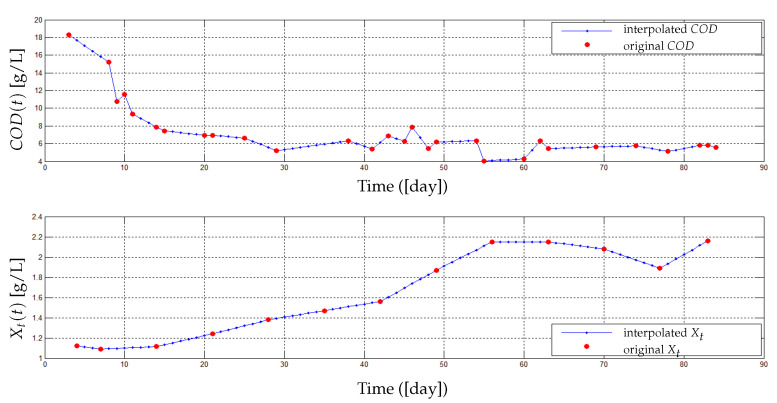
(**Top**): Total COD data in the AnMBR. (**Bottom**): Total biomass data in the AnMBR.

Two cycles of filtration/backwash are considered over 84 days in total: the first cycle for the period [*t* = 1 to 39 days] and the second one for the period [*t* = 41 to 84 days], with a backwash at the 40th day for the first cycle and at the 85th day for the second cycle (Figure 7). Collected data of the total COD ($S_{T}$ and SMP) and the total biomass ($X_{T}$) are represented in Figure 8 (red markers), while data for the permeate flux are shown in Figure 7 (red dots). The initial value of the flux in the first cycle is J(0) = 8.32 L/(h·m^2^) and in the second cycle is J(0) = 8.1 L/(h·m^2^).

### 4.2. Experimental Identification and Validation of the Fouling Model

#### 4.2.1. Parameters Estimation Procedure

In the present case, we apply the least-squares method for the parameter estimation of the models (7)–(11). The objective function (17) is minimized in adjusting model parameters such that the simulated flux $\tilde{J}\left(t\right)$ best fits the experimental flux J(t). A nonlinear optimization algorithm was used (functions “fmincon” and “ode45” of matlab).

(17) $$F=\sum_{i=1}^{N}{\left(J_{i}\left(t\right)-{\tilde{J}}_{i}\left(t\right)\right)}^{2},$$

where *N* is the number of measurements.

Before running the optimization algorithm, we must fix the values of some constants of the model, as the intrinsic resistance membrane $R_{0}$ of Equation (11). It can be estimated from the initial value J(0), which corresponds to the flux obtained with clean water as long as R(t) is still negligible by (18)

(18) $$R_{0}=\frac{\Delta P}{\mu J(0)}.$$

For simplicity, we fixed arbitrarily some parameter values of the models (7)–(11), notably ϵ (0<ϵ<1), σ and $\sigma^{\prime }$ as given in Table 1. Parameters defined in the literature as μ, α and $\alpha^{\prime }$ have default values reported in Table A1.

Model parameters to be estimated by the least-square method are $C_{s}$, $C_{x}$, $C_{smp}$ and $\beta_{1}$. (Since SMPs are the main contributors to pores clogging, they firstly and significantly contribute to pores blocking, thereafter the other solute components can contribute. So, we have chosen $\beta_{2}$ smaller than $\beta_{1}$, for instance $\beta_{2}=\beta_{1}/15$.) They are considered as key parameters used to describe the rate of membrane fouling.

Indeed, in the generic model we have proposed, two sets of parameters are considered: (1) the key parameters to be estimated from experimental data, and (2) the other parameters which are known in the literature or fixed arbitrarily so that we obtain consistent simulations whose best fit with experimental data. For example, we do not exactly know the porous surface area of *A*, but we assume that it could be the majority, so we set ϵ=0.7, which allows us to simulate the case where a membrane has 70% porous surface area. In fact, we did not have enough experimental data to estimate all the model parameters, so we decided to fix the values of some parameters and estimate the values of others.

Our identification procedure consists of using a first part of data of the flux (16 measures for t=1 to 39, see Figure 7 on the left) to estimate parameters $C_{s}$, $C_{x}$, $C_{smp}$ and $\beta_{1}$ and then the last part (18 measures for t=41 to 84, see Figure 7 on the right) to validate the model. Data of total COD ($S_{T}$ and SMP) and total biomass $X_{T}$ (Figure 8) are inserted as inputs for the identification algorithm of the models (7)–(11), such that $S_{T}$ and SMP are assumed to be as follows:

$$S_{T}=85\%COD\;\mathrm{and}\;SMP=15\%COD.$$

Let us emphasize here that this is a simplifying assumption based on what is proposed in the literature, and that in practice, these proportions can change with time and according environmental conditions [11,37,38]. Here, it is just essential that the sum of all soluble matters equals the COD and that the sum of all particulate matters equals the $X_{T}$. Furthermore, we notice that times and frequencies of COD and $X_{T}$ measurements are different (29 measures of COD and only 13 measures of $X_{T}$). To solve this problem, an interpolation of COD and $X_{T}$ at the same times is performed by adding more intermediate points as illustrated in Figure 8 (blue markers). Total interpolated data of 82 points and 80 points are obtained for COD and $X_{T}$, respectively.

#### 4.2.2. Results and Discussion

Simulation and experimental results are plotted in Figure 7. On the left, the simulated flux $\tilde{J}\left(t\right)$ (solid line) is compared with the real measured flux J(t) (red dots) used for calibration. There is a good matching of the model simulations and real data (measures for t=1 to 39), and an accurate correlation coefficient $r^{2}=0.968$ is obtained. On the right, the identified model is validated on the second data set (measures for t=41 to 84), with a satisfactory correlation coefficient $r^{2}=0.938$. Table 2 shows dimensionless parameter values that have been estimated by minimizing the criterion (17).

**Figure 9:**
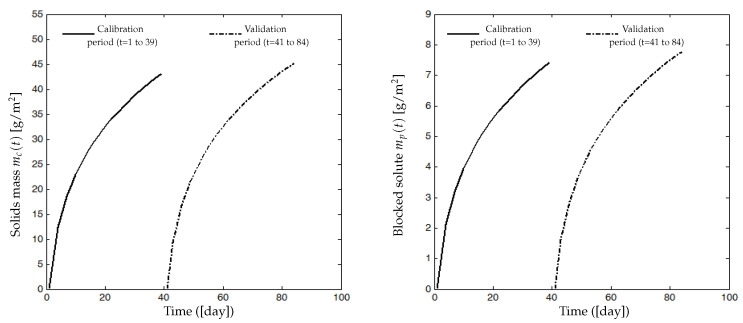
(**Left**): Simulated solid mass $m_{c}\left(t\right)$ attached to the membrane surface. (**Right**): Simulated solute $m_{p}\left(t\right)$ deposited inside the membranes pores.

Figure 9 shows the dynamic evolution of the estimated solids mass $m_{c}\left(t\right)$ attached on the membrane surface and the estimated soluble matter $m_{p}\left(t\right)$ deposited inside the pores. $m_{c}\left(t\right)$ and $m_{p}\left(t\right)$ increase significantly during the first days of the filtration process before their slopes decline when the membrane becomes more and more heavily fouled. At the end of the first filtration period (day 39), the estimated mass $m_{c}\left(t\right)$ is around 43 g/m^2^, and it is about 45 g/m^2^ at the end of the second filtration period (day 84). Estimated quantities of $m_{p}\left(t\right)$ are about 7.4 g/m^2^ and 7.75 g/m^2^ at the end of the first and the second filtration periods, respectively. Moreover, the values of the parameters $m_{c}$ and $m_{p}$ simulated at the end of the second filtration cycle were around 4% higher than the ones simulated at the end of the first cycle. Similarly, the permeate flux simulation at the end of the second cycle (84 days) showed a value of 2.64 L/(h·m^2^) which is lower by about 15% than the value of 3.1 L/(h·m^2^) simulated at the end of the first cycle. As the permeate flux decline is directly related to the increase in the deposited foulant mass $m_{c}$ and $m_{p}$, we assume that the obtained simulations are coherent.

We emphasize that these values are just estimations of $m_{c}\left(t\right)$ and $m_{p}\left(t\right)$ by numerical simulation during the calibration of the models (7)–(11) and that their experimental values may be probably different from those simulated. Of course, if one has experimental measures of $m_{c}\left(t\right)$ and $m_{p}\left(t\right)$, then one can use them to accurately calibrate more parameters of the model.

**Figure 10:**
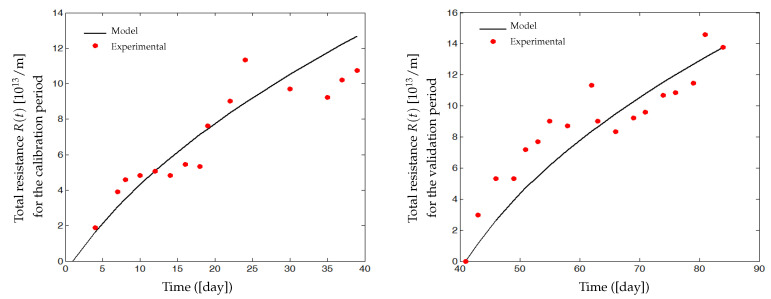
Experimental and simulated resistance R(t) during (**left**): calibration period; (**Right**): validation period.

Figure 10 represents the experimental and the simulated total fouling resistance R(t) due to both $m_{c}\left(t\right)$ and $m_{p}\left(t\right)$. Experimental resistance values are deduced from flux data using Equation (1). Resistance R(t) during the two filtration cycles has the tendency to increase rapidly in the first period, before attaining values of about $12\times 10^{13}$ and $14\times 10^{13}$ at the end of the calibration and the validation period, respectively. If the system is functioning with a long-term filtration, then R(t) should converge towards a quasi-constant value (its equilibrium).

The proposed model is simple from the mathematical point of view, and it reproduces quite well the fouling behavior of the AnBRM: it can then be used for control purposes. However, the estimated parameters values and/or those arbitrarily selected could change with time. Thus, it is necessary to readjust them regularly. For example, one could re-identify on a regular basis parameters in order to best fit the model for the last available measurements (for instance using the last data for t=41 to 84 days, see Figure 7, on the left). Furthermore, if we decide to estimate the parameters of the biological model AM2b, then we will need more informative measurements.

## 5. Discussions, Open Questions and Perspectives on the Process Control Using the Proposed Model

Membrane fouling is the major drawback of MBRs, and one important challenge is to propose new control strategies to minimize fouling and improve treatment efficiency. In particular two important questions must be addressed:

- What parameters mainly influence membrane fouling? This is basically a modeling question.
- How minimizing fouling (filtering conditions) can be seen as a control problem as soon as a model describing the fouling dynamics is available.

If several fouling models have already been proposed in the literature, few were used for minimizing fouling and optimizing treatment. One of them is proposed in [33], where one used a model of SMBR with slow/fast dynamics to develop a nonlinear predictive control. Control of the membrane fouling caused by cake formation in a submerged AnMBR (SAnMBR) was investigated in [2] using biogas sparging. However, very often, the control strategies are tuned heuristically and use available process actuators:

- Gas sparging: It consists of injecting bubbles (air for aerobic process or biogas for anaerobic systems) for membrane scouring in order to limit attachment and promote detachment of matter by shear forces. This control parameter is however costly because it consumes energy. In addition, various parameters of gas sparging as the intensity, the duration and the interval/frequency, may impact on membrane fouling characteristics in the process [39].
- Intermittent filtration: MBR is operated in alternating filtration/relaxation cycles. This functioning mode allows the detachment of matter responsible for the reversible fouling. In [40], the effect of the intermittent filtration combined with gas sparging on membrane fouling in a submerged anaerobic bioreactor was evaluated.
- Backwash: It must be used for a short time compared with the filtration time to detach matter involved in irreversible fouling, and it is costly because it also needs energy. Various scenarios of filtration/backwash for a submerged MBR were investigated in [41] to determine an optimum one. A study in [42] focused on optimizing a backwash frequency, filtration and relaxation strategy for the stable operation of a ceramic tubular AnMBR.

One common question for all these techniques is to find a good control sequence ensuring good process performances while minimizing membrane fouling. In practice, it can be seen as an optimal control problem, since we need to optimize the filtration flux, the filtration time or still the energy consumption. A practical problem could be as follows: what is the optimal sequence for intermittent filtration or for filtration/backwash cycles? What is the optimal operating time and mode for bubbles injection?

A study was performed in [43] with the final aim to reduce by different strategies the energy costs in MBR. In particular, authors investigated the influence of the aeration intensity, the duration of filtration/backwashing cycles, and the number of membrane cleanings on the MBR energy demand. However, the used model is integrated and complicated, which it divided into a biological sub-model (19 biological state variables and 79 parameters) and a physical sub-model (membrane model). In the following, we investigate in simulation the influence of the filtering parameters mentioned above on the flux production and process performances, by using the simple model proposed in this paper.

### 5.1. Gas Sparging Effect

In this section, we investigate how gas sparging can be used for limiting membrane fouling. To do so, we need to modify the proposed models (7)–(11) by adding negative terms on the right sides of Equations (7) and (8). This way, the reversible and irreversible fouling rates are reduced by gas sparging as illustrated by Equations (19) and (20). Functions $f(m_{c})$ and $g(m_{p})$ are positive and represent the controller effect on the detachment of matter (gas sparging, membrane scouring). In some fouling models, these terms are simply constants, but by modulating their magnitude, our idea is to add them here as control parameters (as mentioned above in the modeling section).

(19) $$\begin{array}{ccc} {\dot{m}}_{c} & = & Q_{out}\left(C_{s}S_{T}+C_{x}X_{T}+C_{smp}SMP\right)-f\left(m_{c}\right), \end{array}$$

(20) $$\begin{array}{ccc} {\dot{m}}_{p} & = & Q_{out}\left(\beta_{1}SMP+\beta_{2}S_{T}\right)-g\left(m_{p}\right). \end{array}$$

**Figure 11:**
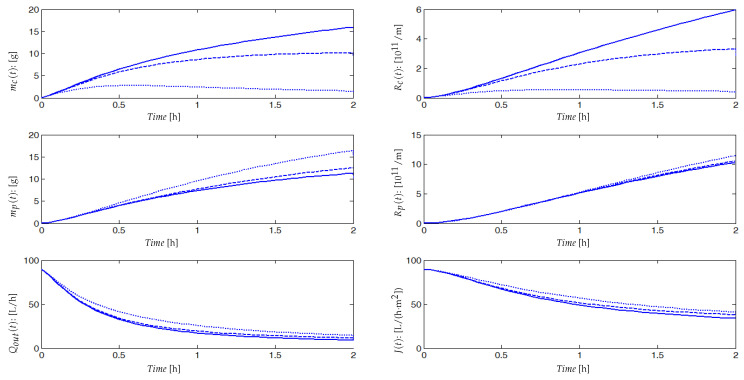
Results simulation of the membrane fouling model with control terms using (19) and (20); (**—**): $k_{m}=0$; (**---**): $k_{m}=0.5$; (⬝⬝⬝⬝⬝): $k_{m}=5$, ($k_{p}=0$).

In other terms, efficient control consists to propose functions $f(m_{c})$ and $g(m_{p})$ depending on the intensity of gas sparging (parameter control). A first simple form of $f(m_{c})$ and $g(m_{p})$ which is already used in the literature is $k_{m}m_{c}$ and $k_{p}m_{p}$, which represent quantities of $m_{c}$ and $m_{p}$ detached by shear forces caused by membrane scouring, where $k_{m}$ and $k_{p}$ depend on the intensity of injected bubbles used to detach fouling [2,44,45,46]. A higher aeration intensity can have a positive effect on cake layer removal by shear force and thus improves the membrane permeability [47]. Figure 11 illustrates the time evolution of the flux J(t) with respect to different values of $k_{m}$ (here, $k_{p}=0$, it is assumed that the irreversible fouling detachment is neglected, since it is not significantly affected by gas sparging). It can be seen that $m_{c}\left(t\right)$ and $R_{c}\left(t\right)$ are inversely proportional to the control parameter $k_{m}$, for higher values of this later, accumulated matter on membrane surface and its corresponding resistance take small values. The output flow $Q_{out}\left(t\right)$ and permeate flux J(t) are increasing proportionally to $k_{m}$. This is a classical result, but the given question is how to optimally calibrate $k_{m}$ (and $k_{p}$) in order to best control the fouling with minimum energy consumption?

On the other hand, one sees in Figure 11 that deposited matters $m_{p}\left(t\right)$ inside the pores and its relative resistance $R_{p}\left(t\right)$ are proportional to $k_{m}$ and inversely proportional to $m_{c}\left(t\right)$. If the value of this parameter increases, then the quantity of $m_{p}\left(t\right)$ and the value of $R_{p}\left(t\right)$ increase likewise leading to a flux loss especially at the end of the filtration time (around steady-state). One can explain this result as follows: if the formed cake layer ($m_{c}\left(t\right)$) represents a second biological membrane (which prevents pores from fouling ($m_{p}\left(t\right)$) as it is known in the literature [29,48,49]) then when this layer is detached by gas sparging, more particles of different sizes go through pores and cause further fouling. So, a second question of such a control strategy may be asked: how can we control and favor the cake formation until acceptable level to protect pores from fouling, but at the same time, without influencing permeate flux? This question actually remains open. The results shown in Figure 11 indicate no considerable effect of decreasing cake formation resistance ($R_{c}$) on the permeate flux which effectively seems to be totally controlled by pore blocking. This situation is usually encountered in the literature, especially when complete pore blocking occurs, the removal of external fouling by increasing the shear rate would not have a considerable effect on permeate flux decline [50,51]. Air sparging seems effectively not useful in the simulated process, and other membrane cleaning process would be more effective such as backwashing or relaxation. 

### 5.2. Influence of the Number of Filtration/Relaxation (Backwash) Cycles per Time Unit

**Figure 12:**
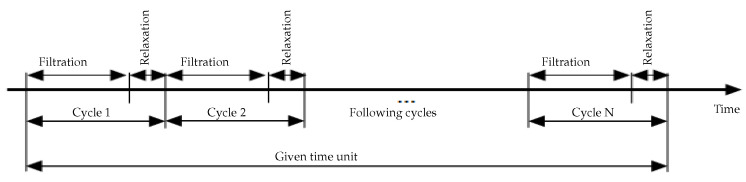
Cycles of filtration/relaxation or backwash per time unit.

Membrane backwashing consists of injecting permeate in the opposite direction to the filtration mode, which allows us to remove physically the foulants blocking the membrane pores as well as the destruction of the cake layer. Moreover, relaxation operation consists of halting the filtration process in order to eliminate the convective forces responsible for the foulant attachment on the membrane surface. This operation would foster the back-diffusion of attached foulants away from the membrane and consequently deconstruct the fouling layer. The effectiveness of those two physical cleaning processes would be higher as cleaning cycles increase. Our objective here is to verify if there is an optimal number of filtration/relaxation or backwashing cycles allowing a higher mean value of MBR output flux. Given a sufficiently large time horizon, what is the optimal number of filtration/relaxation or backwash cycles allowing a higher mean value for the MBR output flux (Figure 12).

**Figure 13:**
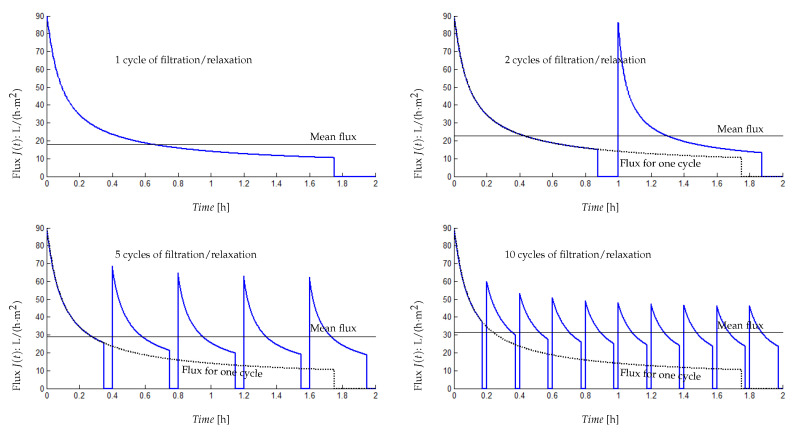
Results simulation of different numbers of filtration/relaxation cycles, with the mean flux (solid horizontal line) on a given functioning period (2 h).

To illustrate the importance of this operational functioning mode, we performed numerical simulations by changing the number of filtration/relaxation cycles over a given functioning period with a constant ratio between filtration time and relaxation time $\alpha_{t}=T_{filtr}/T_{Relax}$ for all cycles. On Figure 13, results are given for one cycle, two cycles, five cycles and ten cycles of filtration/relaxation with a period of 2 h and $\alpha_{t}=7$. We are particularly interested in the mean value $J_{mean}$ of the produced flux on the given period. Using the simulations, we computed the following:

- For 1 cycle of filtration/relaxation, $T_{filtr}=105$ mn, $T_{Relax}=15$ mn and $J_{mean}=17.9$ L/(h·m^2^);
- For 2 cycles of filtration/relaxation, $T_{filtr}=52.2$ mn, $T_{Relax}=7.5$ mn and $J_{mean}=22.9$ L/(h·m^2^);
- for 5 cycles of filtration/relaxation, $T_{filtr}=21$ mn, $T_{Relax}=3$ mn and $J_{mean}=29$ L/(h·m^2^);
- for 10 cycles of filtration/relaxation, $T_{filtr}=10.5$ mn, $T_{Relax}=1.5$ mn and $J_{mean}=31.5$ L/(h·m^2^).

If the objective is to produce a maximum flux over the given period, then 10 filtration/relaxation cycles appears to be the best strategy. As a matter of fact, for a reasonable number of filtration/relaxation cycles and a constant ratio $\alpha_{t}$ between filtration time and relaxation time for all cycles, we have an increasing mean production of flux. However, if the number of intermittent filtration cycles is too large on the considered functioning period, then it can damage the process by forcing it to operate in an On/Off mode with a high frequency. Simulations show that multiplying the number of cycles is beneficial but that this benefit does not increase anymore beyond 10 cycles. Obviously, increasing the number of filtration/relaxation cycles is useful but the higher the frequency, the lower the benefit.

It is thus suggested not to wait too long before proceeding to the membrane cleaning by relaxation or backwash to find the best ratio operated time by benefit in terms of flux produced.

### 5.3. Coupling Gas Sparging and Intermittent Filtration Controls

**Figure 14:**
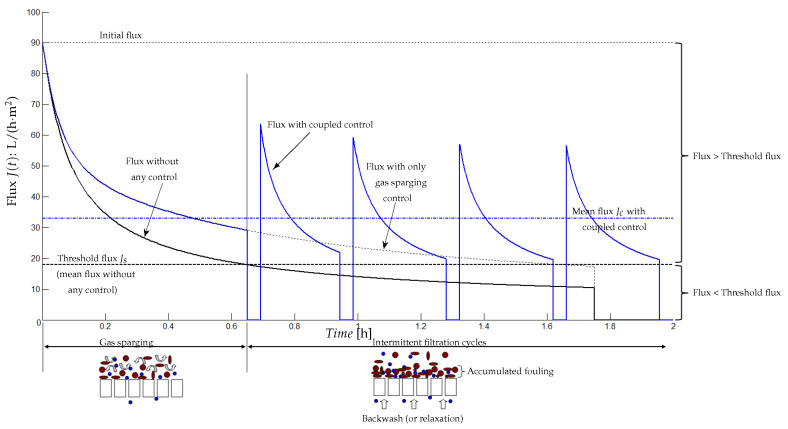
Coupling control based on gas sparging and intermittent filtration.

Figure 14 illustrates a control strategy based on gas sparging which is used at the beginning of the considered period when the flux is still higher than a threshold flux together with intermittent filtration as soon as the flux has reached the threshold flux.

Our idea here is to minimize the energy consumption when using gas sparging and the flux loss (resp. the permeate loss) when the process is in relaxation mode (resp. backwash). In others words, instead of using gas sparging and intermittent filtration simultaneously, we propose to use them sequentially for the following reasons:

- Gas sparging is used to detach the matter deposited on the membrane: this phenomenon occurs at the beginning of the filtration (fouling is soft and not yet dense). Here, one should control the gas sparging intensity, which may depend on different parameters as the mixed liquor characteristics, the concentration of soluble and particulate matters, …
- Intermittent relaxation is used to detach a denser fouling (strong), which can occur after a long enough functioning time. These control parameters (typically the number and frequency of filtration/relaxation (backwash) cycles) may depend on the fouling characteristics as its irreversibility, its thickness…

To illustrate this idea, we performed numerical simulations plotted in Figure 14. The system is first simulated without any control (black line). Then, this reference scenario is compared with the proposed control strategy. It means that gas sparging is first applied until the flux reaches the threshold flux (here $J_{s}=18$ L/(h·m^2^)). At this instant (t=0.64 h), we apply intermittent control with $k_{m}=5$ in Equation (19), where $f\left(m\right)=k_{m}m$ with four cycles.

Simulations show that this control strategy allows one to increase the mean production flux to 33 L/(h·m^2^), whereas the mean flux without control was 18 L/(h·m^2^). As it is noticed in Figure 14, when applying the gas sparging control, it has favorably increased the permeate flux on the control period (until 0.64 h). It should be noticed that even if we applied only the gas sparging all along the functioning period (without using intermittent filtration cycles), the mean flux is 28.76 L/(h·m^2^), lower than the produced flux when the two techniques are used together. Thus, intermittent filtration was an appropriate control strategy to obtain over the whole functioning period of a maximum flux.

Our study on control strategy is obviously in line with other studies such as the work presented in [43]. Their main purpose was to investigate and select the best operating conditions in terms of aeration intensity, duration of filtration/backwashing cycles and number of membrane cleaning to optimize energy demand and operational costs. Thus, interesting further studies may focus on the optimization of the mean production flux, while saving energy consumption and costs. The first results we established here show that this could be achieved by using the simple control model, which integrates biological and membrane compartments of the MBR and evaluating on the considered time unit an appropriate criteria, as a function of the mean production flux, the energy demand and the operational cost. This work is under investigation.

We emphasize that in [52], parameters of the developed models (7)–(11) were identified using data generated by models proposed in [29,33] considered as virtual processes. It was shown that our generic model can capture important properties of these two models, such as the mean value of the transmembrane pressure and the attached mass on the membrane and their dynamics.

In the proposed model, the parameter describing suspended and soluble matter’s rejection by the membrane was not expressed separately. The rejection coefficient of soluble matter was included within the parameters $\beta_{1}$, $\beta_{2}$, $C_{s}$ and $C_{smp}$ expressing the contribution of each soluble matter in cake formation and pore blocking. Moreover, the suspended solids are assumed to be totally rejected by the membrane and would not contribute to the internal fouling of the membrane. In this study, we have not focused on the purity of produced permeate. The objective of this optimization work was mainly to improve the permeate production. The estimation of the soluble matter which crosses the membrane would allow us to estimate the variation in the permeate purity according to the studied operating condition.

## 6. Conclusions

In this paper, we proposed a simple fouling model of anaerobic membrane bioreactor (AnMBR). The model was developed under certain classical hypotheses on the membrane fouling phenomena and was coupled with a reduced order anaerobic digestion model. Two mechanisms of fouling were considered, cake formation on the membrane surface and pores blocking. Contrary to many models of the literature which consider constant membrane surfaces *A*, we assumed that the latter is a decreasing function of both the attached matter $m_{c}\left(t\right)$ and the deposited matter $m_{p}\left(t\right)$ (notably SMP). Our main idea was to introduce in the mass-balance model AM2b [13] a feedback of the decreasing flux due to membrane fouling into the actual output flow rate of the process. We performed simulations to investigate the qualitative behavior of the model, and we validated it on experimental data. It was shown that the proposed model can predict quite well the fouling behavior for the considered AnMBR. It fitted accurately the real flux in both phases of identification: calibration phase and validation phase (see Figure 7). In a second part of the paper, we improved numerical simulations to investigate and discuss the fouling control problem in focusing on the optimal control of gas sparging and intermittent filtration. Preliminary results were obtained about the results of different control strategies over a given time period: at the beginning stage of the process functioning, it appeared useful to use the gas sparging and the intermittent filtration at the end of the considered time period. Based on these results, we proposed to couple control benefits in order to produce the maximum mean flux over the total considered functioning period.

Perspectives of this work include (i) model extension to constant flux and variable TMP filtration and, its validation using other experimental informative data, where parameters identifiability should be studied, (ii) the design of an optimal control to minimize the fouling effects on the system performances while minimizing the energy requirements, (iii) the development of an optimization strategy to control and favor the cake formation until an acceptable level to protect pores from fouling, but at the same time, without influencing permeate flux, and (iv) the coupling of the fouling model with ADM1 model in integrating SMP and possibly EPS (Extracellular Polymeric Substances) dynamics.

## Figures and Tables

**Table 1:** Values of fixed parameters.

Parameter	ϵ	σ	$\sigma^{\prime }$
Value	0.7	10	10
Unit	-	[1/g]	[1/g]

**Table 2:** Values of estimated parameters.

Parameter	$C_{s}$	$C_{x}$	$C_{\mathrm{smp}}$	$\beta_{1}$
Value	0.1970	0.1116	0.9720	0.3999

## Data Availability

Data available on request.

## Appendix A. Parameters Values Used in Simulations

**Table A1:** Parameters values used in simulations.

Parameter	Meaning	Value	Unit	Reference
α	specific resistance of the sludge	$5\times 10^{14}$	[m/kg]	
$\alpha^{\prime }$	specific resistance of the sludge	$1\times 10^{13}$	[m/kg]	
β	SMP fraction leaving the bioreactor	0.6	−	[13]
$\beta_{1}$	SMP fraction blocked into the pores	to be estimated	−	
$\beta_{2}$	$S_{1}$ and $S_{2}$ blocked into the pores	smaller than $\beta_{1}$ (=$\beta_{1}/15$)	−	[13]
σ	parameter to normalize units	10	[g]	
$\sigma^{\prime }$	parameter to normalize units	10	[g]	
μ	the permeate viscosity	0.001	[Pa.s]	
ω	detachment rate of $m_{c}$ during relaxation phase	25	−	
$\omega^{\prime }$	detachment rate of $m_{p}$ during relaxation phase	25	−	
ΔP	transmembrane pressure	1.5	[bar]	[35]
$A_{0}$	initial membrane surface	1	[m^2^]	[35]
$b_{1}$	yield degradation of SMP by $X_{1}$	40	−	[13]
$b_{2}$	yield production of $S_{2}$ from SMP	0.6	−	[13]
$b_{3}$	yield production of SMP from $S_{1}$	3	−	[13]
$b_{4}$	yield production of SMP from $S_{2}$	1.3	−	[13]
$C_{s}$	fraction of $S_{T}=S_{1}+S_{2}$ attached onto the membrane	to be estimated	[1/h]	
$C_{x}$	fraction of $X_{T}=X_{1}+X_{2}$ attached onto the membrane	to be estimated	[1/h]	
$C_{smp}$	fraction of SMP attached onto the membrane	to be estimated	[1/h]	
$C_{s}^{\prime }$	fraction of $S_{1}$ and $S_{2}$ reinjected into the bulk during cleaning operation	0.001	[1/h]	
$C_{x}^{\prime }$	fraction of $X_{1}$ and $X_{2}$ reinjected into the bulk during cleaning operation	0.01	[1/h]	
$C_{smp}^{\prime }$	fraction of SMP reinjected into the bulk during cleaning operation	0.001	[1/h]	
$k_{1}$	yield degradation of $S_{1}$ by $X_{1}$	25	−	[12]
$k_{2}$	yield production of $S_{2}$ from $S_{1}$	15	−	[12]
$k_{3}$	yield degradation of $S_{2}$ by $X_{2}$	16.08	−	[12]
$k_{d1}$	decay rate of the biomass $X_{1}$	0.2	[1/h]	
$k_{d2}$	decay rate of the biomass $X_{2}$	0.18	[1/h]	
$K_{1}$	half-saturation constant associated with $S_{1}$	10	[g/L]	
$K_{2}$	half-saturation constant associated with $S_{2}$	5	[g/L]	
$K_{i}$	inhibition constant associated with $S_{2}$	15	[g/L]	
*K*	half-saturation constant associated with SMP	3	[g/L]	[13]
$m_{1}$	maximum acidogenic biomass growth rate on $S_{1}$	1.2	[1/h]	[12]
$m_{2}$	maximum methanogenic biomass growth rate on $S_{2}$	1.5	[1/h]	[12]
$m_{s}$	maximum acidogenic biomass growth rate on SMP	0.14	[1/h]	[13]
$Q_{in}$	the input flow of the bioreactor	varying	[L/h]	
$Q_{out}$	the output flow of the bioreactor	varying	[L/h]	
$Q_{w}$	the withdraw flow from the bioreactor	1.5	[L/h]	
$R_{0}$	intrinsic membrane resistance	$1.11\times 10^{13}$ (estimated from J(0))	1/m	
$S_{1in}$	the input concentration of $S_{1}$	90	[g/L]	
$S_{2in}$	the input concentration of $S_{2}$	20	[g/L]	
*V*	the volume of the bioreactor	50	[L]	[35]
